# Supplementary material for: Acute Toxic Effects of Tetrodotoxin in Mice via Intramuscular Injection and Oral Gavage
Source: Toxins (Basel). 2023 May 13;15(5):334. doi: 10.3390/toxins15050334 (PMC10220813; doi:10.3390/toxins15050334)
Supplement: Supplementary file 1 [file toxins-15-00334-s001.zip › toxins-2374772-supplementary.pdf]

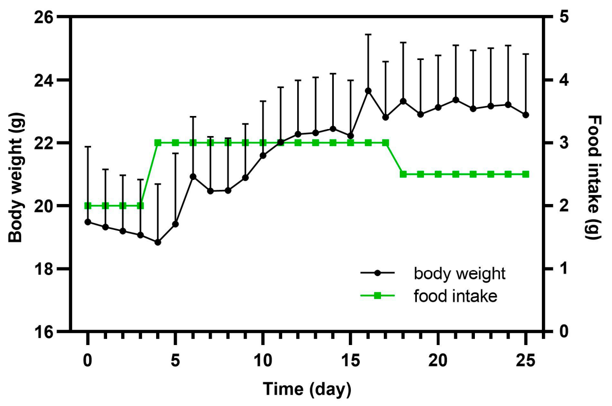

Figure S1. Relationship between daily food intake and body weight in mice ($n = 15$). Male mice were raised with fixed doses of food and free access to water, and their body weights were measured before feeding in the morning. Body weight is expressed as mean ± standard deviation. It was found that the mice lost weight when food intake was maintained at 2 g per day. After maintaining food intake at 3 g per day for 2 weeks, the mice gained 4 g in weight, and the growth trend was approximately consistent with the mice that had free access to food [29]. When we reduced the food intake to 2.5 g per day, the weight of the mice remained at approximately 23 g for 1 week, showing an abnormal growth trend. Thus, we concluded that food intake at 3 g per day met the requirements for normal growth in mice.

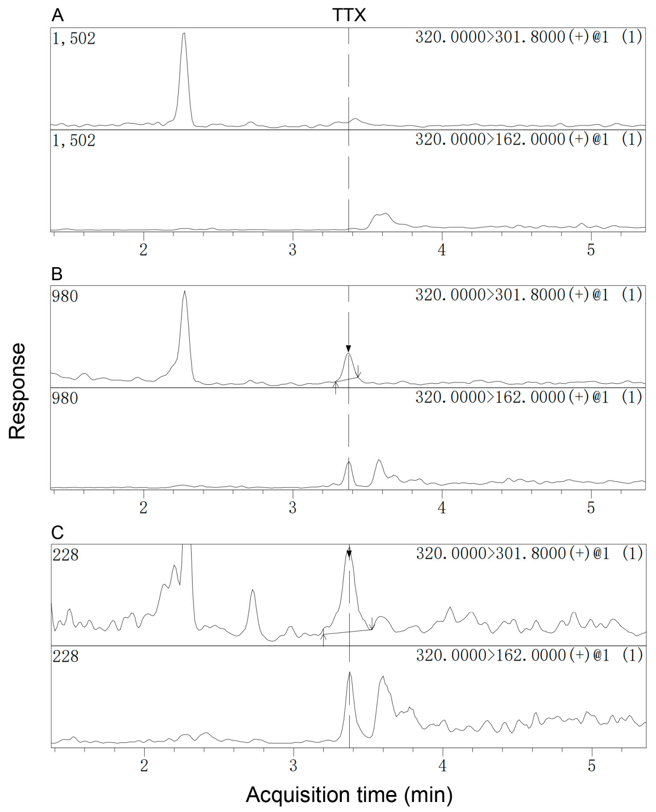

Figure S2. Multiple reaction monitoring chromatograms for ultra-performance liquid chromatography–tandem mass spectrometry analysis of blank serum (A), 4 ng/mL tetrodotoxin (TTX) standard serum (B) and 6.12 ng/mL TTX serum (C, obtained 2 h after TTX o.g. administration) in mice. Ion transitions 320.2 > 301.8/162.0 (*m*/*z*) were used for quantitative and qualitative analysis, respectively.

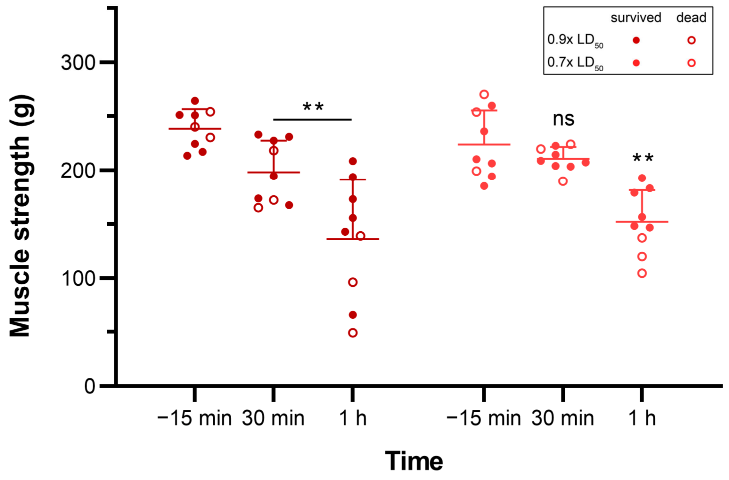

Figure S3. Muscle strength loss in mice after TTX o.g. intoxication within 1 h (n = 9). Data are expressed as mean ± standard deviation. ns, no significance, ** $p < 0.01$ compared with levels before TTX administration (time—−15 min).
